# Attitude of mental healthcare providers toward tele-psychiatry services and associated factors at public referral hospitals in Addis Ababa city, Ethiopia

**DOI:** 10.1186/s13033-023-00596-5

**Published:** 2023-09-12

**Authors:** Jibril Bashir Adem, Mequannent Sharew Melaku, Tirualem Zeleke, Muluken Tesfaye, Firaol Lemessa Kitila, Agmasie Damtew Walle

**Affiliations:** 1 Department of Public Health, College of Health Science, Arsi University, Asela, Ethiopia; 2 Department of Health Informatics, Institute of Public Health, College of Medicine and Health Science, University of Gondar, Gondar, Ethiopia; 3 Department of Psychiatry, Saint Paul’s Hospital Millennium Medical College, Addis Ababa, Ethiopia; 4 Department of Health Informatics, College of Health Science, Mettu University, Mettu, Ethiopia

**Keywords:** Mental health care providers, Attitude, Tele-psychiatry services

## Abstract

**Introduction:**

Health systems around the world are struggling with the massive numbers of people with mental disorders who require professional care. The treatment gap for mental disorders is high all over the world, with between 76 and 85% of people in low- and middle-income countries with severe mental disorders receiving no treatment for their mental health conditions. Tele-psychiatry is used as an alternative solution to the problem of limited mental health services and effective Tele-psychiatry service use may be achievable if mental health providers have a good attitude towards it.

**Objective:**

To assess the attitude of mental healthcare providers toward Tele-psychiatry services and associated factors at public referral hospitals in Addis Ababa city, Ethiopia, 2022.

**Method:**

A Multicenter institution-based cross-sectional study was conducted among 413 mental health professionals working in public referral hospitals in Addis Ababa city, from May 04 to June 10, 2022. Data were collected by using a structured and self-administered questionnaire prepared by reviewing previous related studies. Epi Data version 3.1 and Stata version 14 were used for data entry and analysis respectively. Bivariate and multivariable logistic regression analyses were used to identify factors associated with attitudes toward Tele-psychiatry services. A statistical significance was declared at p-value < 0.05.

**Result:**

A total of 413 Participants were enrolled with a response rate of 91.8%. The majority of respondents 230 (55.69%) were male and the mean age of participants was 29 years (SD + 5.02). In this study the majority (49%) of mental health care professionals had a poor attitude toward Tele-psychiatry. Having electronic health technology experience [AOR 16.79; 95% CI (4.26, 29.3)], lack of training in telemedicine applications [(AOR 0.1; 95% CI (0.01, 0.41)], a good computer uses for daily work activities [AOR 3.65; 95% CI (1.14, 11.60)], availability of e-Health technology awareness program [AOR 0.16; 95% CI (0.03, 0.90)], having a positive perception about the importance of e-Health technologies[AOR 0.041; 95% CI (0.01, 0.29)] and having good knowledge of Tele-psychiatry services [AOR 6.89; 95% CI (1.8, 12.0)] were significantly associated with attitude towards Tele-psychiatry services.

**Conclusion:**

This study found that mental healthcare providers at a public referral hospital in Addis Ababa city generally had poor attitudes regarding Tele-psychiatry services. Considering the significant factors will improve the attitude to use tele-psychiatry services in Ethiopia.

**Supplementary Information:**

The online version contains supplementary material available at 10.1186/s13033-023-00596-5.

## Background

Telemedicine is defined by the World Health Organization (WHO) as “the provision of healthcare services where distance is a critical factor, using information and communication technologies for the exchange of valid information for the diagnosis, treatment, and prevention of disease and injuries; research and continuing education of health care providers [1]. Tele-psychiatry service is one of the most successful forms of telemedicine, which connects people with mental health problems to psychologists and other mental health professionals via telecommunications technology to facilitate effective diagnosis, education, therapeutic interventions, consultation, research, and other medical activities. It has been utilized as an effective solution to the problem of limited psychiatric services for clinics and hospitals in remote areas [2, 3].

Tele-psychiatry service is similar to in-person care in terms of diagnostic accuracy, therapeutic efficacy, and patient satisfaction. It helps to save time, money, and other valuable resources [4]. Tele-psychiatry services are regarded as the most significant use of telemedicine in the Western world for the management and treatment of patients with mental health problems [5]. However, it is still in its infancy in developing countries and functions more as an outgrowth of telemedicine than as a stand-alone service [6]. According to recent research on 114 “WHO” member states focused on telemedicine throughout the world, only 13% of responding nations have developed Tele-psychiatry service programs, and only 24% reported having any Tele-psychiatry services [7].

The global burden of mental diseases has just been reported, revealing a major public health issue that impacts individuals, society, and nations as a whole [8, 9]. An increasing number of people with mental disorders who need professional care is causing health systems around the world to stumble [6]. Between 1990 and 2019, the global number of Disability Adjusted life years (DALYs) due to mental disorders increased from 80·8 to 125·3 million, and an estimated 450 million people worldwide currently have at least one mental disorder [10, 11]. In Ethiopia, the average prevalence of mental disorders was 18% for adults and 15% for children [12]. In addition to this, the populations in the war-affected regions of Afar and Amhara suggest a minimum of 28 560 people with more severe forms of mental health disorders that require immediate intervention, of whom 12 566 are children and 14 565 are women [13].

Despite the significant prevalence of mental disorders in the community and recognition of the resulting disability, which places mental disorders among the top ten major illnesses contributing to disability-adjusted life years, service provision for diagnosis and management is poor [14]. The treatment gap for mental disorders is high all over the world, with between 76 and 85% of people in low- and middle-income countries with severe mental disorders receiving no treatment for their mental health conditions, and the corresponding figures for high-income countries are also high, between 35 and 50% [15]. In developing countries, Tele-psychiatry services are already in significant demand to provide access to specialist services that are otherwise unavailable and to reduce unnecessary travel, particularly in Sub-Saharan African countries [16]. In Ethiopia, there are only two mental health hospitals, and the ratio of psychiatrists to the population is 1 psychiatrist to 1.5 million people [17]. The greatest promise of Tele-psychiatry services is to provide a viable solution to the existing and grossly scarce mental health services [6]. However, there is no standalone tele-psychiatry services in Ethiopia The level of healthcare professionals' knowledge, and perception, their level of skill, their interest, their attitude towards technology, and their work environment are all factors that influence the success of any new technology, for practitioners to accept and provide Tele-psychiatry services, they must receive awareness on the technology and be assessed for their competence to use it [18, 19]. The use of information technology in the healthcare system is influenced by many issues. Among others, human-related components such as users’ knowledge and attitude toward technology are of high importance. A survey in Michigan State University, USA, and other similar studies show that knowledge and attitude is an important and key research question to explain how telemedicine is viewed and conceived by healthcare professionals [20].

Participants were more likely to consider using tele-psychiatry services in the future when using e-Health technology, according to a countrywide multisite survey conducted in the United States. Due to a lack of closeness, there are concerns about the doctor's capacity to identify subtle signs of body language, nonverbal cues, and/or physical signs of disease [21]. The high cost of videoconferencing technology, as well as the necessity for enough bandwidth and the ability to talk in the patient’s native language, has hampered the adoption of tele-psychiatry services in Africa. Tele-psychiatry services from Somaliland to Somali Diaspora communities in Europe have been reported as a pilot study, while there are issues with data security and privacy [22]. A systematic analysis of healthcare professionals’ attitudes toward smartphone applications for depression revealed that, despite some concerns, attitudes toward the use of smartphone applications in clinical practice are generally favourable [23].Despite the critical importance and demand for Tele-psychiatry services in Ethiopia, little is known about mental healthcare providers' attitude towards the use of this care modality for the treatment and management of patients with mental health problems, even though they are commonly the first gatekeepers to telehealth adoption and program success. The objective of this study was to assess mental healthcare providers' attitudes toward the use of Tele-psychiatry and associated factors for the treatment and management of patients with mental health problems in Addis Ababa, Ethiopia.

## Methods

### Study design

A multicenter institutional-based cross-sectional study was conducted to assess the attitude of mental health care providers toward tele-psychiatry services and associated factors at public referral hospitals in Addis Ababa city.

### Study setting

The study was conducted in Ethiopia’s capital, Addis Ababa city. The city is divided into eleven sub-cities and 116 weredas for administrative purposes (The lowest level administrative unit in the city). Addis Ababa now has 54 hospitals, with 14 state-run hospitals, and 40 private hospitals, of which 5 are referral, 3 comprehensives, and 6 of them were general hospitals. There are 98 functional health centers in the city of which 86 are governmental and the rest are owned by NGOs and 534 clinics, with 34 owned by non-governmental organizations. According to MoH National Health Work Update 2019 mental health professionals constitute 0.26% of the national health workforce. There are 111 practicing general psychiatrists; 46 Clinical Psychologist-[MSc]; 10 social workers; 165 Mental Health –MSc; 320 Psychiatry professional-BSc and 111 Psychiatry professional-advance Diploma nationally. Subspecialty wise there are one Forensic, one Addiction and two child and adolescent psychiatrists [24].

### Population

All mental healthcare providers working at public referral hospitals in Addis Ababa city were considered as the source population. Mental health care providers who meet the inclusion criteria were considered as Study Population. Having a minimum of diploma qualification and 6 months or more of work experience were considered as inclusion criteria.

### Sample size determination and sampling procedure

Since no previous study in the same population had been conducted, the sample for this study was calculated using a single population proportion formula [25], with a relative precision of 5%, 10% non-response rate, 95% (CI), and a proportion of mental healthcare providers’ attitude towards Tele-psychiatry service of 50% and it becomes 424.

The study was conducted in four public referral hospitals in Addis Ababa city (St-Paulos Specialized Hospital, Tikur Anbessa Specialized Hospital, Federal Police Referral Hospital, and St-Amanuel mental specialized Hospital). Based on a review of each hospital's human resource list, the total number of mental health care providers in those four hospitals is 450, and the sample size is calculated as 424 using the sample size calculation formula. A survey study was utilized to recruit participants since the calculated sample size is extremely close to the actual number of mental health care professionals in the study setting.

### Variables of study

An outcome variable was the attitude of healthcare professionals toward Tele-psychiatry services. Socio-demographic factors like age, gender, education level, salary, professional types, and work experience, and personal factors like electronic health technology experience, perceptions towards e-Health technology, computer skills, computer use for daily activities, and Internet use for health information access. And organizational factors like access to computers and the internet, the availability of IT support, telemedicine application training, electronic health technology awareness program, and knowledge of mental healthcare providers about Tele-psychiatry services were considered independent variables.

### Operational definition

Tele-psychiatry: is defined as the delivery of mental healthcare services where distance is a critical factor by all healthcare professionals using information and communication technologies for the exchange of valid information for the diagnosis, treatment, and prevention of disease and injuries, research and evaluation, and continuing education of healthcare providers, all in the interest of advancing the health of individuals and their communities.

Mental health professionals are defined as those employees who have at least a diploma in health science and who are practicing mental health care services in their work settings.

Computer Access: the availability of a functional desktop or personal computer to support day-to-day activities in the office or department.

Internet Access: is the availability of any form of active internet connection to support day-to-day activities in the hospital/office/department (Wi-Fi, broadband connection, or others).

Information Communication Technology (ICT) Infrastructures: includes computers, the internet, videoconferencing, power supply, telephone, LCD/projectors, printers/scanners, computer software, CD/Hard disk, and other hardware and software.

Information Technology (IT) supports: In this study, it is information technology maintenance and support given to mental health care providers on the use of information communication technologies. Poor Knowledge of Tele-psychiatry services is defined as a score on the first part of the questionnaire less than 50% and good knowledge of Tele-psychiatry services is defined as a score on the second part of the questionnaire more than 50%. It involves knowing the medical applications of tele psychiatry service, knowing Tele-psychiatry service infrastructure, knowing tele-psychiatry service tools (such as real-time and store and forward Tele-psychiatry service), and knowing the effect of Tele-psychiatry service on the quality of care, cost, and time. Attitude: A mean score of less than 2.5 was considered to indicate a poor attitude, 2.6–3.0 a moderate attitude, and more than 3.0 a good attitude [26].

### Data collection instruments

Data were collected by using a structured and self-administered questionnaire designed for the study. The questionnaire was prepared by reviewing previous related studies [19, 26–32]. The questionnaire was divided into five main sections, including:—Sociodemographic information (six items), organizational information (nine items), and personal information (fourteen items), and knowledge-related information (ten items), and attitude-related information (twenty-three items). Yes-or-no questions were used to assess the respondents’ knowledge of Tele-psychiatry services. “Yes,” answers got a “1,” while “No” answers got a “0.” scores for this section vary from 0 to 9. To determine the degree of Tele-psychiatry knowledge in this study, a cutoff score of 5 (55.56%) on average across the 9 questions was used. A 5-point Likert scale was used to rate the perceived Tele-psychiatry attributes of relative advantage, compatibility, complexity, trial-ability, and observability, except for the complexity attribute questions, which are scored in the opposite direction (1 = strongly agree and 5 = strongly disagree). To calculate the specific mean score, the mean scores for all statements for each perceived Tele-psychiatry quality were added together. In this study, a mean score of less than 2.5 (50%) was considered to indicate a poor attitude, 2.6 (51%) to 3.0 (60%) a moderate attitude, and more than 3.0 (60%) a good attitude.

### Data quality control measures

Data were collected using a pretested, structured, and self-administered questionnaire that was translated into Amharic by researchers and then back to English to ensure that the translated version gives the proper meaning. To assure data quality standardized questionnaires and trained professionals were involved in data collection. The pre-test was done on eleven mental healthcare providers that possessed the same characteristics as study participants at Asella referral and teaching hospital before being provided to study participants, this was done to ensure accuracy or precision of the measuring instrument in terms of clarity, suitability, and flow of questions before the questionnaire was finally administered to participants. Reliability was established by using Cronbach’s alpha calculated using Stata 14 to determine the reliability of all items under the instrument used in this study. Coefficients of between 0.7 and 1.0 are considered good meaning that there is internal consistency. The Cronbach’s alpha reliability coefficient for knowledge-determinant variables (9 items) was 0.84 and 0.96 for attitude-determinant variables (23 items). It was within the acceptable reliability range.

### Data management and analysis

Data were entered into Epi Data version 3.1 and exported to the Stata version 14 software for further analysis. Missing values, outliers, and other inconsistencies were avoided after organizing and exporting data into Stata version 14, and cleaning was done to avoid missing values, outliers, and other discrepancies. Frequency, sorting, and a list were used to clean up the data. Descriptive statistics, including frequencies, means, and proportions were computed and visualized using tables, graphs, and diagrams to describe the data.

To identify factors associated with the attitude of mental healthcare providers toward Tele-psychiatry services, ordinal logistic regression was a reasonable approach to use our data given that all the dependent variables are ordered categorically [33]. However, it’s founded that the proportional odds assumption was violated for all three categories of dependent variables in the preliminary analysis. Therefore, multinomial logistic regression analysis was utilized. Variables were declared statistically and significantly associated with dependent variables at p < 0.05. Moreover, the strength of association between factors and the dependent variables was determined using an Adjusted Odds Ratio (AOR) with a 95% confidence level.

### Ethical consideration

The study was approved by the Research and Ethics Review Committee of the University of Gondar, Public Health Institute (IPH/2120/2014). Our study was performed in accordance with the declaration of Helsinki. Prior to the interviews, all participants provided written informed consent including to participate. Due to the sensitivity of health issues, the ethical guidelines were respected during the whole research process, including keeping respondents anonymous and respecting confidentiality regarding all information given by participants.

## Results

### Socio-demographic characteristics of the study participants

This study was conducted among 450 mental health care providers working in public referral hospitals in Addis Ababa city to assess the attitude of mental health care providers towards tele-psychiatry services. Of the total (450), 413 MHCPs participated with a 91.8% response rate from May 04 to June 10.

The majority (331) of respondents were from St-Amanuel specialized mental Hospital. The majority of respondents 230 (55.69%) were male. The mean age of participants was 28.86 years (SD + 5.02) and the majority 248 (60.05%) of the age group were between 20 and 29 years. A large number of respondents were bachelor’s degree holders 239 (57.87%). Most 142 (34.4%) of the study participants were psychiatry professionals (Table 1).

**Table 1:** Socio-demographic characteristics of mental health care providers working at public referral hospitals in Addis Ababa, Ethiopia

Socio-demographic characteristics	Number (n = 413)	Percent (%)
Gender		
Male	230	55.69
Female	183	44.31
Age		
20–29 years	248	60.05
30–39 years	148	35.84
> 39 years	17	4.12
Educational status		
Diploma	57	13.8
Bachelor degree	239	57.87
Master’s degree	44	10.65
Others	73	17.68
Year of experience		
< 5 years	271	65.62
5–10 years	97	23.49
> 10 years	45	10.90
Type of profession		
Psychiatrist	11	2.66
Psychologist	29	7.02
Psychiatry professional	142	34.38
Social worker	21	5.08
General practitioner	26	6.30
Nurse	101	24.46
Others	83	20.10
Salary		
< 1500 ETB	17	4.12
1500–3500 ETB	27	6.54
3500–5500 ETB	87	21.07
> 5500 ETB	282	68.28

The attitude of healthcare providers toward Tele-psychiatry services

**Fig. 1:**
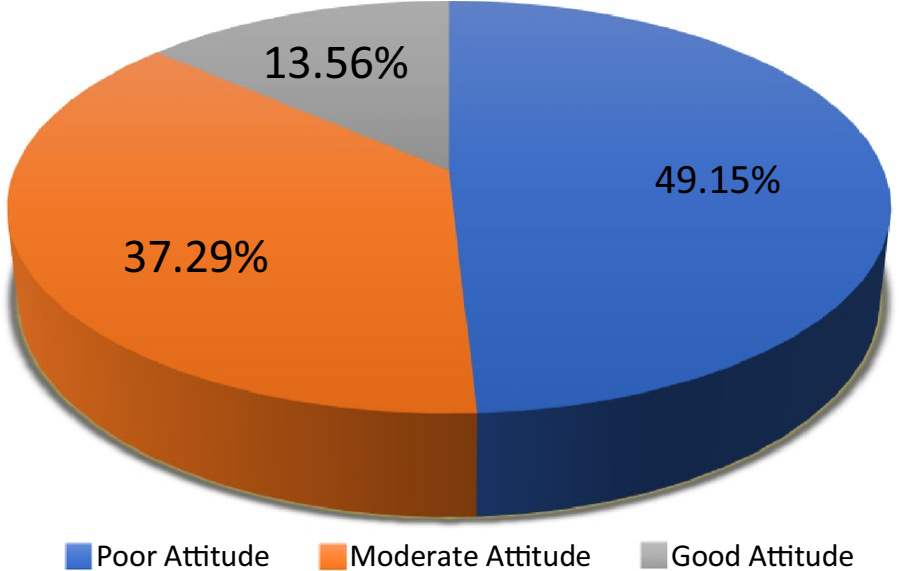
Attitude of mental health care providers toward Tele-psychiatry service

In this study, the majority (49%) of mental health care professionals had a poor attitude toward Tele-psychiatry, with a “relative advantage” mean of 2.68, “observability” mean of 2.64, and “compatibility” means of 2.52, “complexity” means of 2.38 and “trial ability” mean o2.27 (Fig. 1).

Attitudes of mental healthcare providers toward Tele-psychiatry on reducing medical errors, reducing the number of healthcare visits, and improving clinical decisions had a high value, with a “mean of 2.8,” while attitude toward the use of Tele-psychiatry on a trial basis is enough to see what it could do had the low value. With a “mean” of 2.2. Most of the study participants 202 (48.91%) believed that Tele-psychiatry improves communication between healthcare professionals, whereas 187 (45.28%) agreed that Tele-psychiatry decreases the number of visits to healthcare. 87 (23.2%) of the participants believed that Tele-psychiatry increases staff workload and according to 153 (37.05%) of the participants. Tele-psychiatry adds new responsibilities for workers, while 132 respondents (31.96%) strongly agreed that Tele-psychiatry reduces medical error.

The majority of the study participants 206 (49.9%) agreed that Tele-psychiatry facilitates diagnosis and treatments, while 154 respondents (37.05%) agreed that Tele-psychiatry threatens information confidentiality and patient privacy. Only 76 (18.40%) of health professionals believed that Tele-psychiatry is complex, and 87 (21.07%) believed that Tele-psychiatry needs a lot of mental effort. About 82 (19.85%) of study participants thought that Tele-psychiatry should be used for a variety of activities in hospitals, while the majority of study participants, 235 (56.90%), felt that trying Tele-psychiatry applications is a great opportunity for them (Additional file 1: Table S1).

### Factors associated with the attitude of healthcare providers toward tele-psychiatry service

The dependent variable has three categories, all of which are mutually exclusive and exhaustive, there is no multi-collinearity (there is no variable with VIF > 10), the observations are independent, and the dependent variable is assessed at the nominal level. The parallel line test was also used to check the proportional odd assumption, and the P-value was low (P < 0.001), indicating that the proportional odd assumption had been violated. As a result, multinomial logistic regression was applied (Additional file 2).

To determine the factors associated with the attitude toward Tele-psychiatry, a bi-variable analysis was done and computed with each independent variable. In bi-variable analysis at (p < 0.25), respondent’s age, work experience, educational level, computer access, internet access, availability of IT support, training on Tele-psychiatry applications, level of computer skill, perception about eHealth technology, the status of IT support, availability of e-Health technology awareness program, internet use for health information access, source of information about Tele-psychiatry, frequency of internet visit for health information access, computer use for daily work activities, electronic Health(e-Health) technologies experience, knowledge of Tele-psychiatry services, were the variables that showed statistically significant association with the outcome variable that is attitude towards Tele-psychiatry.

In multivariable analysis (multinomial logistic regression) at (p < 0.05), computer use, perception towards e-Health technology, electronic Health(e-Health) technologies experience, availability of e-Health technology awareness program, training in Tele-psychiatry applications, knowledge of Tele-psychiatry services were the variables that showed statically significant association with the outcome variables of moderate attitude towards Tele-psychiatry services, similarly, training on Tele-psychiatry applications, electronic Health(e-Health) technologies experience, perception of electronic health technology, availability eHealth technology awareness program and knowledge of Tele-psychiatry services were showed statically significant association with the outcome variables of good attitudes towards Tele-psychiatry services. Based on McFadden’s result, we might say that the full model containing our predictors represents an 80.16% improvement in fit relative to the null model [LR χ^2^ (50) = 632.95, p < 0.001].

The odds of having a moderate attitude toward Tele-psychiatry services (relative to having a poor attitude) for an individual with good computer use in their daily work activities are estimated to be 3.65 times higher [AOR 3.65; 95% CI (1.14, 11.60] than that of an individual with poor computer use in work activities. When compared to individuals who did not have any experience with electronic health (e-Health) technology, the odds of having a moderate attitude toward Tele-psychiatry services (relative to having a poor attitude) for individuals with eHealth technology experience are anticipated to increase by about 18 units [AOR 17.99; 95% CI (5.43, 30.5)].

For people with negative perceptions about the importance of eHealth technologies. The odds of having a moderate attitude toward Tele-psychiatry services for people with a positive perception about the importance of e-Health technologies (relative to having a poor attitude) are lower by 96% [AOR 0.041; 95% CI (0.01, 0.29)] than it was for people who have a positive perception about the importance of eHealth technologies. Similarly, the odds of having a moderate attitude towards Tele-psychiatry services (relative to a poor attitude) are anticipated to be lower by 0.12 folds for an individual working in the organization without an eHealth technology awareness program [AOR 0.12; 95%CI (0.03, 0.45)] as compared to those individual working in an organization that has eHealth technology awareness program.

Those individuals without any Tele-psychiatry training are expected to have 81% lower odds of having a moderate attitude toward Tele-psychiatry services (relative to having a poor attitude) [AOR 0.19; 95% CI (0.039, 0.88)] than those who have received training in Tele-psychiatry applications. For those individuals with good knowledge about Tele-psychiatry services, the log-odds of having a moderate attitude toward Tele-psychiatry services (relative to having a poor attitude) are estimated to rise by 7.1 units [AOR 7.1; 95 percent CI (1.97, 12.22)] as compared to those with poor knowledge about Tele-psychiatry services.

The odds of having a good attitude toward Tele-psychiatry services (relative to having a poor attitude) for individuals with eHealth technology experiences are estimated to improve by 16.79 times when compared to people who did not have experience with electronic health (e-Health) technologies (AOR 16.79; 95% CI (4.3, 29.3)]. For people who have a negative perception towards e-Health technologies, the odds of having a moderate attitude towards Tele-psychiatry services (relative to having a poor attitude) are lower by 0.07 units [AOR 0.07; 95% CI (0.01, 0.55)] than it was for people who have a positive perception towards e-Health technologies.

The odds of having a good attitude toward Tele-psychiatry services (relative to a poor attitude) are anticipated to be lower by 0.16 folds for an individual working in the organization without an eHealth technology awareness program [AOR 0.16; 95% CI (0.03, 0.90)] as compared to those individual working in an organization that has eHealth technology awareness program.

Those of individual who did not receive any training in Tele-psychiatry applications are expected to have 90% lower odds of having a good attitude toward Tele-psychiatry services (relative to having a poor attitude) [AOR 0.1; 95% CI (0.01, 0.41)] than those who have received training in Tele-psychiatry applications. The odds of having a good attitude toward Tele-psychiatry services (relative to having a poor attitude) are predicted to increase by 6.89 units for those with good knowledge of Tele-psychiatry services compared to people with low knowledge of Tele-psychiatry services [AOR 6.89; 95% CI (1.8 12.0)] (Table 2).

**Table 2:** Factors associated with an attitude of healthcare providers toward Tele-psychiatry services

Poor attitude (reference)		Attitudes of healthcare providers (number = 413)			COR (95% CI)	AOR (95% CI)
Moderate attitude	Variables	Poor	Moderate	Good		
	Age					
	> = 29 years	108	108	32	1	1
	30–39 years	80	39	29	0.48(0.30, 0.77)	0.81 (0.002, 4.47)
	< = 40 years	9	3	5	0.33(0.1, 1.26)	4.32 (0.01, 12.5)
	Training on Tele-psychiatry applications					
	Yes	22	110	52	1	1
	No	175	40	14	0.045 (0.03, 0.81)	0.19** (0.039, 0.88)**
	E-health awareness					
	Yes	22	137	56	1	1
	No	175	13	10	0.01(0.006, 0.024)	0.12**(0.03, 0.45)**
	Perception towards e-health technology					
	Positive	91	147	58	1	1
	Negative	106	3	8	0.02(0.005, 0.057)	0.041**(0.01, 0.29)**
	Knowledge of Tele-psychiatry					
	Poor	134	70	39	1	1
	Good	63	80	27	2.43 (1.57, 3.77)	7.1** (1.97, 12.22)**
	Computer use					
	Poor	43	139	59	1	1
	Good	154	11	7	0.02 (0.01, 0.04)	3.65** (1.14, 11.60)**
	E-Health technologies experience					
	Yes	93	46	35	0.49 (0.32, 0.77)	17.99** (5.43, 30.55)**
	No	104	104	31	1	1

Good attitude												
	Age											
	> = 29 years	108	108	32	108	108	32	32	108	32	1	1
	30–39 years	80	29	3	39	29	3	29	29	3	1.22 (0.68, 2.18)	0.67 (0.01, 3.72)
	< = 40 years	9	29	5	3	29	5	5	29	5	1.87 (0.58, 5.99)	4.2 (0.07, 21.12)
	Computer use											
	Poor	43			139			59			1	1
	Good	154			11			7			0.03 (0.014, 0.78)	2.89(0.82, 10.20)
	E-Health technology awareness program											
	Yes	22			137			56			1	1
	No	175			13			10			0.02(0.01, 0.05)	**0.16 (0.03, 0.90)**
	E-health technologies experience											
	Yes	93			46			35			1.26(1.12, 2.21)	**16.79 (4.3, 29.3)**
	No	104			104			31			1	1
	Training in Tele-psychiatry applications											
	Yes	22			110			52				
	No	175			40			14				
	Perception towards e-health technology											
	Positive	91			147			58				
	Negative	10			3			8				
	Knowledge of Tele-psychiatry services											
	Poor	134			70			39			1	1
	Good	63			80			27			1.47 (1.04, 2.62)	**6.89 (1.8, 12.0)**

Bold shows the significance of the variables at 95% CI

## Discussion

The purpose of this study was to assess the attitude of health professionals toward Tele-psychiatry services and factors associated with it, at public referral hospitals in Addis Ababa city. In this study, majority (49%) of mental health care professionals had a poor attitude toward tele-psychiatry. Before moving forward with real technology acquisition and deployment, management may need to make sure that favorable knowledge and a positive collective attitude have been created and maintained among healthcare practitioners regarding telemedicine and the services it offers [34]. Effective Tele-psychiatry service use may be achievable if mental health practitioners have good knowledge and attitudes toward it and are skilled in using information and communication technology tools [35].

The proportion of health care providers with a good attitude toward Tele-psychiatry in this study was about 14% [95% CI (11%, 17%)], which is lower than the result of the study among Spanish psychiatrists 62% [36], the findings of an Indian study 63% [37], and the findings of studies on the attitudes of Syrian health care providers that (75%) had a favorable attitude toward Tele-psychiatry [38]. This discrepancy may be due to the difference in sample size (152 participants in Spain, 102 participants in India, and 33 participants in Syria’s study), usage of a semi-structured questionnaire, and study population of post-graduate trainee and post-degree working residents in India’s study.

In this study, computer use, perceptions toward e-Health technology, experience with e-Health technology tools, and knowledge of tele-psychiatry services, and access to training on Tele-psychiatry applications were the factors that affected healthcare professionals’ attitudes toward Tele-psychiatry services.

In this study, the odds of having a good attitude toward Tele-psychiatry services for those who have experience in electronic health technology tools are predicted to increase by about 17 folds as compared to those of participants with no experience in electronic health (e-Health) technologies tools. Studies show that healthcare professionals prefer in-person visits and may even prefer in-person therapy, but after using telemedicine technologies, their perceptions of it improved, suggesting that more exposure for clinicians may be needed to allay their concerns about rapport and to enhance their attitude toward those technologies [39]. The findings of an Indian study also emphasize the importance of public awareness campaigns, health professional training, and the establishment of hospital training programs for all clinicians, all of which will aid in the future use of telemedicine applications [40].

The results of this study show that individuals who have a negative perception about the significance of eHealth technologies have odds of having a good attitude toward Tele-psychiatry services that are 93% lower than individuals who have a positive perception. This shows that the overall perception of healthcare providers about the importance of electronic health technology in healthcare needs to be improved before moving to the real application of any telemedicine applications. Healthcare administrators need to start preparing their personnel for the upcoming technology improvements that will occur in their organizations.

While there are many obstacles standing in the way of the widespread use of electronic health technologies, those related to physicians are not intractable. On the contrary, providers and healthcare institutions can accelerate physician acceptance and, eventually, the rate of adoption and utilization by methodically assessing physicians' perceptions of electronic health technology. The ability to offer targeted education to highlight the benefits of electronic health technologies and further improve physician perceptions of electronic health technologies will be made possible by an understanding of physician perceptions [41].

The finding of this study suggests that those healthcare providers who received training in Tele-psychiatry applications are predicted to have 10 times higher odds of having a good attitude toward Tele-psychiatry services than those who did not receive any training. This result is consistent with the study conducted in Karachi, which found that the majority of clinicians had poor attitudes towards telemedicine applications and that a large percentage (98.2%) of them complained that their workplaces had no telemedicine training or workshops, which prevented them from being familiar with telemedicine guiding principles. They claimed that to stay current with the most recent telemedicine technologies, professionals needed ongoing training. The necessity for telemedicine technology development, research, and support is well acknowledged among clinicians [42].

The results of this study reveal that individuals who work in organizations with eHealth technology awareness programs will have about 6 times higher odds of having a good attitude toward Tele-psychiatry services as compared to people working in organizations without e-Health technology awareness programs. This may be because a person’s perception and use of innovative health technologies can be enhanced by having a better understanding of electronic health technology. Telemedicine by its nature highly associated with a lot of ethical issues. Issues of major concern are related to the security and confidentiality of patient data and so appropriate awareness creation and training in telemedicine ethics and medico-legal issues in telemedicine would solve this issue [40].

In this study, it is projected that those who have a good knowledge of Tele-psychiatry services have 7.1 times higher odds of having a moderate attitude toward Tele-psychiatry services and 6.9 times higher odds of having a good attitude toward Tele-psychiatry services. This may be since the degree to which health professionals are aware of and understand the concept, and the skills they have acquired, are all elements that affect how providers of health care feel about technology and the success of any new health technology. This is true for all evolving medical technologies, including Tele-psychiatry services, where practitioners need to be educated on the technology and evaluated for their suitability to accept and offer Tele-psychiatry services [18, 19].

## Limitations and strengths of the study

Due to the lack of research on the topic, it’s difficult to discover sufficient studies to compare and contrast the findings with those of others, which made the discussion shallow. This study was conducted among health professionals working in public referral hospitals in Addis Ababa city, as a result, this finding may not be attributed to the whole healthcare professional population. It would be more useful and generalizable if this study was conducted in the whole country to determine the attitude of a larger sample of health professionals in more facilities and regions than we were able to cover.

The study assessed attitude, therefore the results may not correspond to what people actually do. Despite these shortcomings, the findings of this study offer important insight into the knowledge and attitudes of mental healthcare professionals towards Tele-psychiatry services and their associated factors.

### Recommendations

The Ethiopian Federal Ministry of Health (FMOH) and stakeholders working on mental healthcare programs should facilitate e-health technology awareness programs and training and telemedicine services to improve the healthcare providers’ knowledge and attitude towards Tele-psychiatry services. The EFMOH should also ensure the supply of computers, network, and internet services to boost ICT infrastructure for the proper functioning of all electronic health technologies. Additionally, the Ethiopian Federal Ministry of Health should avail updated and revised policies and guidelines on the initiations and use of electronic health technologies for improving knowledge and attitude health care providers towards Tele-psychiatry.

Furthermore, health facilities should improve the healthcare provider’s use of electronic health technologies in their daily work activities. Health facilities should also facilitate health care providers training on computer skill to improve their computer use in daily work activities. Health facilities could ensure the supply of computers, network or internet services, telemedicine technology instruments, and avail ICT infrastructure for the proper functioning of all electronic health technologies. Finally, further research will be needed to assess the overall knowledge and attitudes of healthcare providers towards tele-psychiatry services including public and private health facilities across the country.

## Conclusion

This study found that, generally, mental health care professionals had poor attitudes toward Tele-psychiatry services. In this study, attitudes toward Tele-psychiatry services were found to be associated with good computer use for daily work activities, eHealth technology experiences, and positive perceptions of e-health technologies, e-Health technology awareness programs, training in telemedicine applications, and having good knowledge about Tele-psychiatry services.

### Supplementary Information

**Additional file 1: Table S1.** Attitude of mental health care providers towards attributes of Tele psychiatry services.

**Additional file 2: Table S1.** Socio-demographic related questions. **Table S2.** Organizational related questions. **Table S3.** Personal related questions. **Table S4.** Knowledge Related Questions. **Table S5.** Attitude Related Questions.

## Data Availability

The datasets used and/or analyzed during the current study will be available from the corresponding author upon reasonable request.

## Abbreviations

APA: American psychiatric association
DALY: Disability adjusted life years
EMOH: Ethiopian federal ministry of health
HIPAA: Health insurance portability and accountability act
ICT: Information communication technology
IT: Information technology
MHCP: Mental health care provider
VoIP: Voice over internet protocols
WHO: World Health Organization
