# Supplementary material for: Attitude of mental healthcare providers toward tele-psychiatry services and associated factors at public referral hospitals in Addis Ababa city, Ethiopia
Source: Int J Ment Health Syst. 2023 Sep 12;17:26. doi: 10.1186/s13033-023-00596-5 (PMC10496341; doi:10.1186/s13033-023-00596-5)
Supplement: Supplementary file 1 — Additional file 1: Table S1. Attitude of mental health care providers towards attributes of Tele psychiatry services. [file 13033_2023_596_MOESM1_ESM.docx]

**Table S1 Attitude of mental health care providers towards attributes of Tele psychiatry services**

| Attributes of Tele psychiatry attitude | Strongly disagree | Disagree | Neutral | Agree | Strongly Agree | Mean score |
| --- | --- | --- | --- | --- | --- | --- |
| **Relative Advantage** | | | | | | |
| Reduce medical errors | 16(3.9%) | 46(11.1%) | 53(12.8%) | 166(40.2%) | 132(31.9%) | 2.8 |
| Facilitate diagnosis and treatment | 8(1.9%) | 31(7.5%) | 46(11.2%) | 206(49.9%) | 122(29.9%) | 2.54 |
| Increase communication among health care providers | 7(4.1%) | 31(17.4%) | 33(22.3%) | 202(34.9%) | 140(21.3%) | 2.64 |
| reduce the number of visits to healthcare centers | 8(1.9%) | 30(7.3%) | 35(8.5%) | 187(45.3%) | 153(37.1%) | 2.8 |
| Enables me accomplish my task more quickly | 8(1.9%) | 29(7.0%) | 57(13.8%) | 199(48.2%) | 120(29.1%) | 2.6 |
| Improve clinical decisions | 13(3.1%) | 46(11.1%) | 83(20.1%) | 148(35.8%) | 123(29.8%) | 2.8 |
| Provide more comprehensive healthcare services | 5(1.2%) | 44(10.7%) | 71(17.2%) | 176(42.6%) | 117(28.3%) | 2.62 |
| **Total mean score 2.67** | | | | | | |
| **Compatibility** | | | | | | |
| In my opinion, Tele psychiatry is compatible with all aspects of my work | 152(37%) | 74(17.9%) | 90(21.8%) | 152(36.8%) | 82(19.9%) | 2.52 |
| Tele psychiatry is completely compatible with my current situation | 17(4.1%) | 72(17.4%) | 92(22.3%) | 144(34.9%) | 88(21.3%) | 2.59 |
| I think Tele psychiatry fits well with the way I like to work | 180(1.4%) | 61(14.8%) | 74(17.9%) | 180(43.6%) | 92(22.3%) | 2.44 |
| Using Tele psychiatry fits well into my work style | 10(2.4%) | 72(17.4%) | 83(20.1%) | 158(38.3%) | 90(21.8%) | 2.52 |
| **Total mean score 2.52** | | | | | | |
| **Complexity** | | | | | | |
| I believe using Tele psychiatry requires a lot of effort | 25(6.1%) | 170(41.2%) | 65(15.1%) | 87(21.1%) | 66(15.9%) | 2.57 |
| Learning to operate Tele psychiatry is hard for me | 29(7.1%) | 256(61.9%) | 50(12.1%) | 61(14.8%) | 17(4.1%) | 2.24 |
| I think Tele psychiatry Increase staff work load | 37(8.9%) | 256(55.9%) | 50(11.9%) | 70(16.9%) | 17(6.3%) | 2.32 |
| I think Tele psychiatry Create new responsibilities for staff | 20(4.8%) | 109(26.4) | 73(17.7%) | 153(37.1%) | 58(14.1%) | 2.32 |
| In my opinion, Tele psychiatry Threaten information confidentiality and patient privacy | 30(7.3%) | 93(22.5%) | 59(14.3%) | 154(37.3%) | 77(18.6%) | 2.47 |
| **Total mean score 2.38** | | | | | | |
| **Trial ability** | | | | | | |
| I believe to try Tele psychiatry applications is a great opportunity | 8(1.9%) | 23(5.6%) | 47(11.4%) | 235(56.9%) | 100(24.2%) | 2.31 |
| I do not have to take very much effort to try out Tele psychiatry | 21(5.1%) | 59(14.3%) | 78(18.9%) | 183(44.4%) | 21(17.4%) | 2.33 |
| I believe, using Tele psychiatry on a trial basis is enough to see what it could do | 13(3.2%) | 47(11.4%) | 51(12.4%) | 225(54.5%) | 77(18.6%) | 2.2 |
| I would like to try out Tele psychiatry applications before using it | 9(2.2%) | 54(13.1%) | 41(9.9%) | 224(53.8%) | 87(21.1%) | 2.237 |
| **Total mean score 2.27** | | | | | | |
| **Observability** | | | | | | |
| In the hospital, I see Tele psychiatry being used for many tasks | 45(10.9%) | 170(41.2%) | 43(10.4%) | 81(19.6%) | 74(17.9%) | 2.663 |
| Tele psychiatry is very visible in the hospital where I work | 51(12.4%) | 184(44.6%) | 29(7.0%) | 82(19.9%) | 67(16.2%) | 2.605 |
| I have seen what other hospital staffs do with Tele psychiatry | 52(12.6%) | 177(42.9%) | 26(6.3%) | 82(19.8%) | 76(18.4%) | 2.668 |
| **Total mean score 2.645** | | | | | | |
| **Overall attitude means score 0.644** | | | | | | |
