# Supplementary material for: Attitude of mental healthcare providers toward tele-psychiatry services and associated factors at public referral hospitals in Addis Ababa city, Ethiopia
Source: Int J Ment Health Syst. 2023 Sep 12;17:26. doi: 10.1186/s13033-023-00596-5 (PMC10496341; doi:10.1186/s13033-023-00596-5)
Supplement: Supplementary file 2 — Additional file 2: Table S1. Socio-demographic related questions. Table S2. Organizational related questions. Table S3. Personal related questions. Table S4. Knowledge Related Questions. Table S5. Attitude Related Questions. [file 13033_2023_596_MOESM2_ESM.zip › Supplementary Files/Amaharic translation.docx]

 University of Gondar

College of Medicine and Health Sciences

Institute of Public Health

**የመረጃ ወረቀት**

**ርዕስ**:ስለ ቴሌሳይካትሪ አገልግሎት እውቀት እና አመለካከት እና በአእምሮ ጤና አጠባበቅ አቅራቢዎች መካከል በአዲስ አበባ ከተማ ውስጥ ባሉ የህዝብ referral እና specialized ሆስፒታሎች ውስጥ ያለው ተያያዥ ምክንያቶች ፣ 2022 ፣ ኢትዮጵያ

ስሜ __________________ እባላለው ጅብሪል በሽር አደም ወክዬ ነው። በጎንደር ዩኒቨርሲቲ የህብረተሰብ ጤና ትምህርት ኢንፎርማቲክስ ኢንስቲትዩት የማስተርስ ሁለተኛ ዲግሪ ተማሪ ሲሆን የማስተርስ ድግሪውን በከፊል መሟላቱን የአእምሮ ጤና ባለሙያዎች ስለ ቴሌሳይካትሪ አገልግሎት ያለው እውቀት እና አመለካከት እና ተያያዥ ምክንያቶች በህዝብ ሪፈራል እና ስፔሻላይዝድ ላይ ምርምር እያደረገ ነው. ስለ ጥናቱ እና የጥናቱ ተሳታፊ ለእርስዎ ለማስረዳት ትኩረትዎን እንዲሰጡኝ በአክብሮት እጠይቃለሁ. የዚህ ጥናት ዋና ዓላማ በሕዝብ ጤና ላይ የማስተርስ ዲግሪ ከፊል ሙሌት ሆኖ ተሲስ መጻፍ ነው። ይህንን ጥናት ለማካሄድ ከጎንደር ዩኒቨርሲቲ የስነምግባር ክሊራንስ አለኝ። በዚህ ጥናት ላይ ለመሳተፍ ተመርጠዋል እና የማይታወቁ መልሶችዎ ለምርምር ዓላማዎች ብቻ ጥቅም ላይ ይውላሉ. የጥናቱን ዓላማ በብቃት ለመድረስ ለእያንዳንዱ ጥያቄ እውነተኛ ምላሽ እንድትሰጡ እጠይቃለሁ። እንዲሞሉ የሚፈልጓቸው ጥያቄዎች አሉ እና በመጠይቁ ላይ ስምዎን ማስቀመጥ አያስፈልግም። የእርስዎ መልሶች ሙሉ በሙሉ ሚስጥራዊ ናቸው። እምቢ ማለት፣ ማንኛውንም ወይም ሁሉንም ጥያቄዎች ለመመለስ ሙሉ መብትህ ነው። መሳተፍ ካልፈለጉ መጠይቁን ባዶ መተው ይችላሉ።ጥናቱ በጎንደር ዩኒቨርሲቲ ተቀባይነት አግኝቷል።

የፍቃድ የምስክር ወረቀት

የተሳታፊ ኮድ _____________

የዚህ ጥናት ግኝቶች ለሆስፒታል አስተዳደር እና ውሳኔ ሰጪዎች እንደሚተላለፉ ተረድቻለሁ ይህም ለቀጣይ የቴሌሳይካትሪ ህክምና አገልግሎት የአእምሮ ጤና ችግር ላለባቸው ታማሚዎች ህክምና እና አስተዳደር በግብአትነት ይጠቅማል።

በዚህ ጥናት ለመሳተፍ በፈቃዴ ተስማምቻለሁ።

የተሳታፊዉ ፊርማ ። ________________ ቀን__________________

የጠያቂው ስም፡- ____________________ ፊርማ ____________ ቀን_______

በጥናቱ ዙሪያ ጥያቄ ካሎት በሚከተለው አድራሻ ስልክ ቁጥር 0922836268 ወይም

Email: adamjibrilbashir@gmail.com

**መጠይቅ**

የመጠይቅ/ ኮድ፡ ________________

**መመሪያዎች**

**ይህ መጠይቅ የአእምሮ ጤና ባለሙያዎች ስለ ቴሌሳይካትሪ አገልግሎት እውቀት እና አመለካከት ለመገምገም ነው። ይህ ጥናት በጎንደር ዩኒቨርሲቲ የምርምር ስነ ምግባር ኮሚቴ ፀድቋል። በዚህ ጥናት ላይ ለመሳተፍ በዘፈቀደ ተመርጠዋል እና ተሳትፎዎ በፍላጎትዎ ላይ ብቻ የተመሰረተ ነው። በዚህ ጥናት ውስጥ ላለመሳተፍ የመምረጥ መብት አለዎት እና በማንኛውም ጊዜ ለማቆም መብት አለዎት. ሁሉም መረጃ በስምነት ይመዘገባል ስለዚህ እባክዎን ስምዎን ወይም ማንኛውንም መታወቂያ አይጻፉ**

- ጠያቂው አምስት ክፍል ያለው ሲሆን መጠይቁን ለመሙላት ከ10-15 ደቂቃ ያህል ይወስዳል።
- እባካችሁ ጥያቄዎቹን በእውነት መልሱ።
- እባክዎ ሁሉንም ጥያቄዎች ይመልሱ።

እባክዎን በተዘጋጀው ሳጥን ውስጥ ምልክት ያድርጉበት፣ ትክክለኛ መልሶችን በተሰጡት ባዶ ቦታዎች ላይ ክብ ያድርጉ።

የመረጃ መሰብሰቢያ ቀን፡- ________________

የሆስፒታሎች ኮድ: _____________

**ክፍል - A**

**የአእምሮ ጤና ባለሙያዎች ስነ-ሕዝብ መረጃ**

1. ዕድሜ በዓመታት ________
2. ፆታ

A. ወንድ

B. ሴት

1. የስራ ልምድ

A. ከ1 አመት በታች

B. 1-5 ዓመታት

C. 6-10 ዓመታት

D.11-15 ዓመታት

E. ከ 15 ዓመት በላይ

1. የትምህርት ደረጃ

A. የምስክር ወረቀት

B. ዲፕሎማ

C. የመጃማረ ዲግሪ

D. የሕክምና ዶክተር

E.ማስተርስ ዲግሪ

F. ፒኤችዲ

1. የሥራ ርዕስ / ሚና

A. የስነ-አእምሮ ሐኪም

B. ሳይኮሎጂስት

C. ነርስ

D. ማህበራዊ ሰራተኛ

E. የአእምሮ ህክምና ነርስ

F. አማካሪ

G. ሐኪም (ከአእምሮ ሐኪም በስተቀር)

H. የየህብረተሰብ ጤና ባለሙያ

I. ሌላ

**ክፍል - B**

**ድርጅታዊ ምክንያቶች**

1. **የኮምፒተሮች መረጃ**

1. በዲፓርትመንትዎ/ቢሮዎ ውስጥ የኮምፒውተር አለዎት?

A) አዎ

B) አይ

1. **የበይነመረብ መረጃ**

1. በዲፓርትመንትዎ/ቢሮዎ ውስጥ የኢንተርኔት አገልግሎት አሎት?

A) አዎ

B) አይ

2. ለጥያቄ ቁጥር 1 አዎ ከሆነ፣ በእርስዎ ክፍሎች/ቢሮዎች ውስጥ ያለውን የኢንተርኔት ጥንካሬ እንዴት ይገመግማሉ

A) በጣም ጥሩ

B) ጥሩ

C) ደካማ

D) በጣም ደካማ

1. **የአይሲቲ መሳሪያዎች መገኘት**

1. በእርስዎ ክፍሎች/ቢሮ/ተቋማት ውስጥ ICT መሳሪያዎች አሉ?

A. አዎ

B. አይ

2. "አዎ" ከሆነ ለጥያቄ ቁጥር 1፣ ከሚከተሉት መሳሪያዎች ውስጥ አሁን ባለህበት ቦታ በቀላሉ ማግኘት የምትችለው የትኛው ነው? (ብዙ መልሶች ሊኖሩ ይችላሉ).

A) የቪዲዮ ኮንፈረንስ

B) ፓውረ ሰፐለይ

C) ስልክ

D) LCD / ፕሮጀክተሮች

E) አታሚ / ስካነር

F) የኮምፒውተር ሶፍትዌር

G) ሲዲ / ሃርድ ዲስክ

H) ሌሎች/እነሱን ጥቀስ። ____, ______,____,________

1. **የአይቲ ድጋፍ መገኘት**

*1.*ከድርጅቶችዎ የአይቲ ድጋፍ አለ?

A. አዎ

B. አይ

*2.*ለጥያቄ ቁጥር 1 አዎ ከሆነ፣ ከተቋማትዎ/መምሪያዎ ያገኙትን የአይቲ ድጋፍ እንዴት ይገመግማሉ?

A) በጣም ጥሩ

B) ጥሩ

C) ደካማ

1. **የኤሌክትሮኒክስ ጤና (E-Health) ቴክኖሎጂዎች ግንዛቤ**

1. በድርጅትዎ/በክፍልዎ ውስጥ የeHealth ቴክኖሎጂ ግንዛቤ ፕሮግራም አለ?

A) አዎ B) አይ

2. ለጥያቄ 1 አዎ ብለው ከመለሱ፣ ምን ያህል ጊዜ የግንዛቤ ፕሮግራም ተቀብለዋል።?

A) አንድ ጊዜ

B) ሁለት ጊዜ

C) ሦስት ጊዜ

D) አራት ጊዜ እና ከዚያ በላይ

1. **በቴሌሳይካትሪ መተግበሪያዎች ላይ ስልጠና**

1.በየትኛውም የቴሌፕሳይኪያትሪ አፕሊኬሽን ሰልጥኖ ታውቃለህ??

A) አዎ B) አይ

2. አዎ ከሆነ ለጥያቄ ቁጥር 1 በየትኞቹ የቴሌፕሳይኪያትሪ አገልግሎት ዘርፎች ተቀብለዋል? (ብዙ መልስ ማግኘት ይቻላል)

A) የቪዲዮ ኮንፈረንስ

B) የቴሌፕሳይካትሪ መመሪያዎች

C) የጽሑፍ መልእክት ላይ የተመሠረተ የታካሚ ማማከር እና ሕክምና

D) የስልክ ጥሪ ማማከር እና ክትትል

E) ኢንተርኔት ላይ የተመሰረተ ምክክር እና ክትትል

F) በኢሜል ላይ የተመሰረተ የታካሚ ማማከር እና ክትትል

G) ከቴሌፕሲካትሪ ጋር የተያያዙ የስነምግባር ጉዳዮች

H) ሌላ/ጠቅሷቸው____፣ _____፣ ________

**ክፍል - C**

**የግል ምክንያቶች**

**A**. የኮምፒውተር ችሎታ

1. የኮምፒውተር ችሎታ አለህ

A.አዎ

B. አይ

2. ለጥያቄ ቁጥር 1 አዎ ከሆነ የኮምፒተርዎን ችሎታ እንዴት ይገመግማሉ

A. ጀማሪ

B. አማካኝ

C. ባለሙያ

**B. የኤሌክትሮኒክስ ጤና (e-Health) ቴክኖሎጂዎች ልምድ**

1. የኤሌክትሮኒክ የጤና ቴክኖሎጂዎችን ማንኛውንም መተግበሪያ ተጠቅመህ ታውቃለህ

A. አዎ

B. አይ

2. ለጥያቄ ቁጥር 1 አዎ ከሆነ፣ የትኞቹን የኤሌክትሮኒክስ የጤና ቴክኖሎጂዎች ተጠቅመዋል?

A.ኤሌክትሮኒክ የሕክምና መዝገብ

B. የሞባይል ጤና

C. ተለባሽ የጤና መሣሪያ

D. ቴሌሄልዝ

E. ቴሌሜዲኬሽን

F. ኤሌክትሮኒክ የጤና መዝገብ

G. የግል የጤና መዝገብ

H. ሌላ፣ ጥቀሳቸው__፣ _____፣ _____

**C**. **ስለ ኤሌክትሮኒክ ጤና( e-Health) ቴክኖሎጂዎች አስፈላጊነት ግንዛቤ**

1. በዕለት ተዕለት የጤና አጠባበቅ አሰጣጥ እንቅስቃሴዎች ውስጥ የኤሌክትሮኒክስ ጤና ቴክኖሎጂዎች አስፈላጊ ናቸው ብለው ያስባሉ

A.አዎ

B. አይ

2. ለጥያቄ ቁጥር 1 አዎ ከሆነ፣ የኤሌክትሮኒክስ ጤና ቴክኖሎጂ በጤና አገልግሎት አሰጣጥ ሥርዓት ውስጥ ያለውን ጠቀሜታ እንዴት ይገመግማሉ?

A. በጣም አስፈላጊ

B. አስፈላጊ

C. ብዙ አስፈላጊ አይደለም

D. የበይነመረብ አጠቃቀም ለጤና መረጃ ተደራሽነት

1. በበይነመረብ ላይ የሕክምና መስክ ጎብኝተው ያውቃሉ?

A.አዎ

B. አይ

2. ለጥያቄ ቁጥር 1 አዎ ከሆነ፣ የጤና መረጃ ለማግኘት ምን ያህል ጊዜ ኢንተርኔት ይጠቀማሉ

A.ሁሌም

B. ብዙ ጊዜ

C. አንዳንድ ጊዜ

D. አልፎ አልፎ

**ኢ የኮምፒውተር አጠቃቀም**

ከታች ላሉት ለእያንዳንዱ ጥያቄዎች፣የእርስዎን አጠቃቀም በተሻለ ሁኔታ የሚለይበት ቦታ ላይ እባክዎ ምልክት ያድርጉ 🗸

| NO. | ጥያቄዎች | በጭራሽ | አልፎ አልፎ | በየሳምንቱ | በየቀኑ | በቀን ውስጥ ብዙ ጊዜ |
| --- | --- | --- | --- | --- | --- | --- |
| 1 | በሥራ ቦታ ኮምፒተርን ምን ያህል ጊዜ ይጠቀማሉ? |  |  |  |  |  |
| 2 | በቤት ውስጥ ኮምፒተርን ምን ያህል ጊዜ ይጠቀማሉ? |  |  |  |  |  |
| 3 | ከጤና ጋር የተያያዙ መረጃዎችን በበይነመረብ (online) ላይ ለመፈለግ ስንት ጊዜ ኮምፒተርን ይጠቀማሉ? |  |  |  |  |  |
| 4 | ከጤና በላሞያ ጋር ለመገናኘት ምን ያህል ጊዜ ኢ-ሜይልን ይጠቀማሉ? |  |  |  |  |  |
| 5 | በበይነመረብ (online) ላይ ስለ እርስዎን ማነጋገር ዘዴዎች በታካሚዎች ተጠይቀዋል? |  |  |  |  |  |
| 6 | በበይነመረብ በኩል ምን ያህል ጊዜ መረጃን ያወርዳሉ/ ይሰቅላሉ? |  |  |  |  |  |

Table 3: የኮምፒውተር አጠቃቀም

**ክፍል ዲ**

**ከእውቀት ጋር የተያያዙ ጥያቄዎች**

ከታች ላሉት ለእያንዳንዱ ጥያቄዎች፣ መልሱን በተሻለ በሚያሳይዎት ቦታ ላይእባክዎ ምልክት ያድርጉ

| አይ | **ጥያቄ** | **አዎ** | **አይ** |
| --- | --- | --- | --- |
| 1 | ስለ ቴሌሳይካትሪ ሰምተህ ታውቃለህ? |  |  |
| 2 | አዎ ከሆነ፣ የመረጃ ምንጭዎ ምን ነበር? | ስልጠና {______}  የህዝብ ሚዲያ {______}  ኢንተርኔት {______}  የስራ ባልደረቦች {______}  ሌሎች/ጠቅሷቸው______፣ ______፣ | |
| 3 | ከዚህ ቀደም የቴሌፕሳይኪያትሪ ሂደት አይተህ ታውቃለህ? |  |  |
| 4 | እንደ REAL TIME (የቪዲዮ ኮንፈረንስ) እና ማከማቻ እና ማስተላለፍ (የጽሑፍ መልእክት ፣ ኢሜል እና የመሳሰሉት) ስለ ቴሌሳይካትሪ መተግበሪያዎች ያውቃሉ? |  |  |
| 5 | ቴሌሳይካትሪ በጤና አጠባበቅ ጥራት ላይ ስላለው ተጽእኖ ያውቃሉ? |  |  |
| 6 | ቴሌሳይካትሪ በአእምሮ ጤና አገልግሎት አቅርቦት እና በእነርሱ ፍላጎት መካከል ያለውን ልዩነት ይቀንሳል? |  |  |
| 7 | ስለ ቴሌሳይካትሪ መሠረተ ልማት ታውቃለህ? |  |  |
| 8 | አላስፈላጊ የመጓጓዣ ወጪን በመቀነስ ላይ ስለ ቴሌሳይካትሪ ጥቅሞች ያውቃሉ? |  |  |
| 9 | የክሊኒኩን ጊዜ በመቆጠብ ረገድ ቴሌሳይካትሪ ስላለው ጥቅም ያውቃሉ? |  |  |
| 10 | ስለ ቴሌሳይካትሪ መመሪያዎች ያውቃሉ |  |  |

**TABLE 4: ከእውቀት ጋር የተያያዙ ጥያቄዎች**

**ክፍል - E**

**ከአመለካከት ጋር የተያያዙ ጥያቄዎች**

ከታች ላሉት ለእያንዳንዱ ጥያቄዎች፣ መልስዎን በተሻለ ሁኔታ በሚያሳየው ምርጫ ስር ባለው ክፍት ቦታ ላይእባክዎ ምልክት 🗸 ያድርጉ ።

(1= በጣም እስማማለሁ….5= በጣም አልስማማም)

| N**O** | **ቴሌሳይካትሪ ይችላል ብዬ አምናለሁ….** | በጠንካራ ሁኔታ  አልስማማም | አልስማማም | | ገለልተኛ | እስማማለሁ | በጣም እስማማለሁ |
| --- | --- | --- | --- | --- | --- | --- | --- |
| A | **አንጻራዊ ጥቅሞች** |  |  |  | |  |  |
| 1 | የሕክምና ስህተቶችን ይቀንሱ |  |  |  | |  |  |
| 2 | ምርመራ እና ህክምናን ማመቻቸት |  |  |  | |  |  |
| 3 | በጤና ባለሙያዎች መካከል ግንኙነትን ይጨምራል |  |  |  | |  |  |
| 4 | ወደ ጤና አጠባበቅ ማዕከላት የሚደረጉትን ጉብኝቶች ቁጥር ይቀነሳል |  |  |  | |  |  |
| 5 | ስራዬን በበለጠ ፍጥነት እንድፈጽም ያስችለኛል። |  |  |  | |  |  |
| 6 | ክሊኒካዊ ውሳኔዎችን ያሻሽላል |  |  |  | |  |  |
| 7 | የበለጠ አጠቃላይ የጤና አገልግሎቶችን ይስጠል |  |  |  | |  |  |
| **B** | **ተኳኋኝነት** |  |  |  | |  |  |
| 8 | በእኔ አስተያየት ቴሌሳይካትሪ ከሁሉም የሥራዬ ገጽታዎች ጋር ተኳሃኝ ነው |  |  |  | |  |  |
| 9 | ቴሌሳይካትሪ አሁን ካለኝ ሁኔታ ጋር ሙሉ በሙሉ ይስማማል። |  |  |  | |  |  |
| 10 | እኔ እንደማስበው ቴሌሳይካትሪ መስራት ከምወደው መንገድ ጋር በጥሩ ሁኔታ የሚስማማ ይመስለኛል |  |  |  | |  |  |
| 11 | ቴሌሳይካትሪን መጠቀም ከስራዬ ጋር በጥሩ ሁኔታ ይጣጣማል |  |  |  | |  |  |
| **C** | **ውስብስብነት** |  |  |  | |  |  |
| 12 | ቴሌሳይካትሪን መጠቀም ብዙ ጥረት እንደሚያስፈልግ አምናለሁ። |  |  |  | |  |  |
| 13 | ቴሌሳይካትሪን መስራት መማር ለእኔ ከባድ ነው። |  |  |  | |  |  |
| 14 | እኔ እንደማስበው ቴሌሳይኪያትሪ የሰራተኞችን የስራ ጫና ይጨምራል |  |  |  | |  |  |
| 15 | እኔ እንደማስበው ቴሌሳይካትሪ ለሠራተኞች አዲስ ኃላፊነቶችን ይፈጥራል |  |  |  | |  |  |
| 16 | በእኔ አስተያየት ቴሌሳይካትሪ የመረጃ ሚስጥራዊነት እና የታካሚ ግላዊነትን አስፈራራ |  |  |  | |  |  |
| D | **የሙከራ ችሎታ** |  |  |  | |  |  |
| 17 | የቴሌሳይካትሪ መተግበሪያዎችን መሞከር ትልቅ እድል ነው ብዬ አምናለሁ። |  |  |  | |  |  |
| 18 | ቴሌሳይኪያትሪን ለመሞከር ብዙ ጥረት ማድረግ የለብኝም። |  |  |  | |  |  |
| 19 | አምናለሁ፣ ቴሌሳይኪያትሪን በሙከራ ደረጃ መጠቀም ምን ሊያደርግ እንደሚችል ለማየት በቂ ነው። |  |  |  | |  |  |
| 20 | ከመጠቀምዎ በፊት የቴሌፕሳይኪያትሪ መተግበሪያዎችን መሞከር እፈልጋለሁ |  |  |  | |  |  |
| ኢ | **ታዛቢነት** |  |  |  | |  |  |
| 21 | በሆስፒታል ውስጥ ቴሌሳይኪያትሪ ለብዙ ተግባራት ሲውል አይቻለሁ |  |  |  | |  |  |
| 22 | እኔ በምሠራበት ሆስፒታል ቴሌፕሳይኪያትሪ በጣም ይታያል |  |  |  | |  |  |
| 23 | ሌሎች የሆስፒታል ሰራተኞች በቴሌፕሳይኪያትሪ የሚያደርጉትን አይቻለሁ |  |  |  | |  |  |

**Table 5: ከአመለካከት ጋር የተያያዙ ጥያቄዎች**

**ለጥያቄዎቼ መልስ ለመስጠት ጊዜ ስለወሰድክ አመሰግናለሁ፣ እርዳታህን አደንቃለሁ!!!**
